# Dealing With Negative Workplace Gossip: From the Perspective of Face

**DOI:** 10.3389/fpsyg.2021.629376

**Published:** 2021-06-03

**Authors:** Boqiang Zong, Shiyong Xu, Lihua Zhang, Jinzhao Qu

**Affiliations:** ^1^Department of Human Resource Management, School of Labor and Human Resources, Renmin University of China, Beijing, China; ^2^Center for Human Resource Development and Assessment, School of Labor and Human Resources, Renmin University of China, Beijing, China

**Keywords:** negative workplace gossip, gossip, face, self-monitoring, affective events theory

## Abstract

In this study, we investigate the coping response of individuals who are being gossiped about. Drawing on face research and affective events theory, we propose that employees who are targets of negative gossip will actively respond to the gossip about them via engagement in negative gossip themselves. The findings showed that negative workplace gossip stimulated fear of losing face and led to subsequent behavioral responses, namely, engaging in negative gossip. Moreover, self-monitoring, as a moderating mechanism, mitigated the negative impacts of negative workplace gossip on the targets. We discuss theoretical implications for gossip research and note its important practical implications.

## Introduction

For most people in an organization, it is inevitable to become the target of negative gossip. Negative gossip refers to informal communications with other members (i.e., the receiver) about a negative behavior or characteristics of a third party who is absent at work (Brady et al., 2017). A growing number of studies have suggested that negative gossip can have a detrimental effect on the targets. For example, recent empirical studies have shown that negative gossip can have destructive effects on emotional well-being, e.g., emotional exhaustion (Wu X. et al., 2018; Liu et al., 2020), other-directed emotional responses (e.g., Martinescu et al., 2019a,b), cognitions [organization-based self-esteem (e.g., Wu L. Z. et al., 2018)], behaviors [proactive behavior (e.g., Wu X. et al., 2018; Martinescu et al., 2021)], and creative behavior (e.g., Liu et al., 2020) of targeted individuals. Clearly, how these targets deal with negative gossip about them is a subject worth addressing. However, although previous studies have demonstrated the behavioral consequences of being the targets of gossip (e.g., Feinberg et al., 2014; Wu L. Z. et al., 2018; Martinescu et al., 2019a; Dores Cruz et al., 2020), very little attention has been paid to the counterattack of the targets in order to reduce the detrimental effects of negative gossip within organizations.

To fill this gap, this study combines face research with affective events theory (AET, Weiss and Cropanzano, 1996) in an attempt to reveal the emotional consequences of negative gossip in the workplace and corresponding behavioral responses. “Face” refers to “an image of self, delineated in terms of approved social attributes” (Goffman, 1967, p. 5). To a large extent, face, on which people depend to survive in their positions, is built on a variety of foundations, such as reputation, competence, and performance. Therefore, people are generally worried about losing face in the workplace (Brown, 1970). Based on this, this study links negative workplace gossip with face and resulting behavioral responses for the following reasons: first, negative workplace gossip contains information that denigrates the performance and ability in the role of the targets, which could exacerbate their fear of losing face (Gluckman, 1963; Grosser et al., 2010). Second, face research has shown that people worry about their external images related to their positions if threatened, and that these face concerns can prompt them to engage in face-saving behaviors (Goffman, 1955; Harrison et al., 2018; Martinescu et al., 2019a,b). As a result, we suggest that negative gossip may cause the targets to feel fear of losing face and react accordingly.

In short, by combining face research with AET (Weiss and Cropanzano, 1996), we propose a new theoretical framework to understand the emotional impacts of negative gossip in the workplace on targets and their subsequent behavioral responses. Specifically, we identify fear of losing face as a mediating mechanism in the relationship between negative workplace gossip and engaging in negative gossip. We suggest that negative gossip can arouse fear of losing face in the targets (Zhang et al., 2011), and then trigger the targets to participate in negative gossip to ease their worries. According to AET, personality traits can influence the process of emotional response (Weiss and Cropanzano, 1996). Additionally, some studies have shown that individuals respond differently to face their threats, because personality characteristics play an important role in this process (Ho, 1976). Self-monitoring as a trait may play this role, that is, it may affect how the targets react to the gossip about them. Self-monitoring is defined as the extent to which individuals are willing and able to control their public expression and shape their public appearances under the guidance of social appropriateness (Snyder, 1979). Thus, given the importance of self-monitoring in making sense of and dealing with information related to humiliation or embarrassment and external image (Turnley and Bolino, 2001), we identify it as a construct that refers to the extent to which individuals are willing and able to control their public expression and shape their public images (Snyder, 1979). We predict that self-monitoring moderates the relationship between negative workplace gossip and fear of losing face.

This study aims to make several theoretical contributions to the literature. First, our study extends the previous research on negative workplace gossip by addressing how the targets deal with negative gossip, and by introducing a novel face-based mechanism in this process, namely, fear of losing face, as a focal mediating mechanism, which is explicated in detail. Second, we contribute to the workplace gossip literature by integrating it with face research for the first time. Although scholars studying gossip often explicitly or implicitly mention its impact on external images (Wu et al., 2016; Tassiello et al., 2018), empirical research is still scarce. In this regard, we introduce a specific concept related to face, fear of losing face, and theorize and empirically test the connection between the two concepts. Finally, we introduce a moderating mechanism for workplace gossip, namely, self-monitoring.

## Theory and Hypothesis Development

### Negative Workplace Gossip and Engaging in Negative Gossip

Gossip is ubiquitous in organizations. Recently, it has been defined as “a sender communicating to a receiver about a target who is absent or unaware of the content” (Dores Cruz et al., 2021). Typical gossip can either be positive or negative (Brady et al., 2017). Compared with positive gossip, within an organization, negative gossip is generally considered to have a greater impact on the targets (Wert and Salovey, 2004). After all, “good news travels slowly and bad news has wings.” In the workplace, negative gossip can provide performance-related information, such as poor performance and/or disapproval of the behavior of the targets, which is highly detrimental to the reputation of the targets, as reputation is necessary for their career advancement and development in the organization (Bell, 2003). In addition, negative gossip can be used as a tool to reshape organizational norms. For example, through negative gossip, gossipers could emphasize to the audience the legitimacy of their own norms and make them widely accepted in the group, which helps maintain their images in the position (Foster, 2004; Shaw et al., 2011).

Negative gossip, an evaluative conversation that communicates reputation information, makes the target aware of murmurs about his/her position, criticism of his/her abilities, and even potential damage to his/her external image (Brady et al., 2017), which can be regarded as an affective event (Wu X. et al., 2018). We posit that engaging in negative gossip may be the response of the targets to the negative gossip about them. Gossip has multiple social functions. First, gossiping, as a means of emotional venting and coping, can help relieve the anxiety and stress generated by affective events (Brady et al., 2017; Dores Cruz et al., 2019a). Second, gossiping can be used as a tool for information collection to make sense of the real and actual situations in order to gain insight into the norms of the group. This can help the target reduce the uncertainty created by negative gossip events and take appropriate actions, such as justifying themselves (Eder and Enke, 1991; Beersma and Van Kleef, 2012; Brady et al., 2017). Finally, gossiping is a tool for reshaping organizational norm, allowing gossipers to enforce their own norms, thereby strengthening the legitimacy of their positions and maintaining a positive image of themselves (Noon and Delbridge, 1993; Shank et al., 2019). Also, other studies support the reasoning. For example, research on aggression has suggested that indirect forms of aggression, such as gossiping, are more likely to be used by people than direct forms when manipulating reputation. This is because direct aggression is more obvious and easier to be detected by the targets than an indirect one. That is, it is easy to expose identity or hostile intentions, making perpetrators of direct aggression more likely to be confronted with retaliation from the targets than the perpetrators of indirect aggression (Archer and Coyne, 2005). Studies on negative reciprocity have also shown that the targets will take action against the perpetrators, referred to as “an eye for an eye” (Andersson and Pearson, 1999; Greco et al., 2019). Thus, we suggest that the targets of gossip themselves have a tendency to engage in negative gossip in response to being targeted by gossipers.

Therefore, we propose the following:

H1. Negative workplace gossip is positively related to engaging in negative gossip of the targets.

### Negative Workplace Gossip and Fear of Losing Face

Face refers to “an image of self, delineated in terms of approved social attributes” (Goffman, 1967, p. 5). In social interactions, actors can gain face by acting in accordance with the norms of their roles (Kim and Nam, 1998; Tuncel et al., 2020). Actors lose face when they deviate from socially acceptable norms, fail to live up to the expectations of their audience, fail to fully perform their position, and/or are not liked by others (Miron-Spektor et al., 2015; Bourgoin and Harvey, 2018). Actors use face-work, a variety of verbal and non-verbal tactics, to convey the information that improves their face and resist face-threatening events (Ho, 1976; Bourgoin and Harvey, 2018). It is worth noting that face is different from status (Ho, 1976). Status refers to the relative position of a person within a group, for example, which can be obtained through association with other prominent figures (Benjamin and Podolny, 1999), whereas face refers to performing well in social roles. Thus, face is a more inclusive and extensive concept that can be built on and derived from status or other relevant concepts (Ho, 1976). Although the term “face” originally came from China, the central tenet of face is universal (Ho, 1976). Indeed, in social encounters, it is universal to pursue a good reputation and/or a positive self image in the eyes of others (Ho, 1976; Cupach and Metts, 1994). Several studies have shown that face also exists in Western culture (e.g., Mak et al., 2009; Liu et al., 2012; Miron-Spektor et al., 2015).

Fear of losing face reflects the concern of an individual with a disapproving image and/or negative evaluations, in terms of their performance in their position (Zhang et al., 2011). In the workplace, face has positive external benefits, such as supervisor performance ratings (Wayne and Ferris, 1990; Huang et al., 2013), pay increase (Bartol and Martin, 1990), and promotions (Liu et al., 2020), and it is also a part of the identity of someone (Harrison et al., 2018). Therefore, these inform us that we should avoid losing face in specific positions.

The AET clarifies that workplace events can trigger the emotional responses of individuals (Weiss and Cropanzano, 1996). Negative workplace gossip, a negative, evaluative talk that involves important information about the poor performance and/or disapproved behaviors of the target in the position, can leave the target worried about damaging his/her reputation and external image in the position, that is, fear of losing face. Therefore, people in the workplace believe that negative gossip about them means that they are already performing poorly in their position, which leads to a negative external image in the eyes of others and ultimately aggravates the fear of losing face. Thus, we propose the following hypothesis:

H2. Negative workplace gossip is positively related to fear of losing face.

### Fear of Losing Face and Engaging in Negative Gossip

Fear of losing face can trigger behavioral responses in individuals to avoid disapproval or negative evaluation (Zhang et al., 2011). Many previous studies on gossip provide a functional account for gossiping in organizations. Engagement in negative gossip can be a good way for gossipers to preserve their own image through reinforcing the norms (e.g., McAndrew et al., 2007; Watson, 2011; Beersma and Van Kleef, 2012; Brady et al., 2017; Hess and Hagen, 2019; Archer and Coyne 2005). In this regard, we posit that gossiping may be an effective way to deal with the fear of losing face. Specifically, gossiping, as a norm-setting behavior, can help to shift the norms in line with the interests of the gossipers and re-establish their competence in the positions they occupy (Brislin, 1980; Baumeister et al., 2004), which helps them meet or even exceed the expectations in the eyes of others, create good external images, and thus alleviate the fear of losing face (Ho, 1976; Zane and Yeh, 2002). Also, gossiping can be a means of emotional venting and coping. Prior research, for example, has suggested that gossiping may help relieve the negative emotions of the gossipers (Brislin, 1980; Brady et al., 2017; Dores Cruz et al., 2019a). Taken together, we believe that people who fear of losing face may be motivated to engage in negative gossiping to address face concerns. Therefore, we propose the following:

H3. Fear of losing face is positively related to engaging in negative gossip.

H4. Fear of losing face mediates the positive indirect relationship between negative workplace gossip and engagement in negative gossip of the target.

### The Moderating Effect of Self-Monitoring

Self-monitoring is defined as the extent to which individuals are willing and able to control their public expression and shape their public appearances under the guidance of social appropriateness (Snyder, 1979). High self-monitors, according to social appropriateness, are more willing and proficient in modifying their social images in line with the situational demands and role expectations of others; whereas low self-monitors express behaviors that are less controlled by deliberate attempts to behave in situation-appropriate ways (Snyder, 1979; Kudret et al., 2019). Specifically, similar to social chameleons, high self-monitors have almost all of the requisites and sufficient skills to successfully mold and tailor their self-presentation in line with situational appropriateness (Snyder, 1979; Snyder and Gangestad, 1982, 1986). A series of studies have shown that high self-monitors are attentive to information related to their external images and that, at the same time, they have great skills in controlling the images they present to others (e.g., Turnley and Bolino, 2001; Smart Richman and Leary, 2009; Bolino et al., 2016; Wu et al., 2021). Although high self-monitors are more likely to pay attention to the reputation information around them, they are quite skilled and confident in the management of their own reputation. For example, Harrison et al. (1996) demonstrated that people with a high level of self-monitoring showed higher self-efficacy and better adaptability in their interpersonal communication. In addition, research on social networks has shown that high self-monitors have more instrumental and friendship ties. The former can help to effectively obtain important work information to ease uncertainty, and the latter can help individuals to form friendships to ease emotions (Garland and Beard, 1979; Oh and Kilduff, 2008; Borgatti and Halgin, 2011; Kilduff and Lee, 2020). Therefore, these findings indicate that high self-monitors may be better at dealing with negative gossip and avoiding being perceived as less competent or desirable in their positions, which is to say they are not afraid of losing face.

Therefore, based on the AET, which points out that personality traits influence the process of workplace events that affect emotional responses and subsequent behaviors (Weiss and Cropanzano, 1996), we propose that people who are high self-monitors are less likely to consider negative gossip as a worrying face-threatening event because they are always aware of and alert to negative information around them and they have the confidence to deal with it. Hence, a high level of self-monitoring reduces the damaging impact of negative workplace gossip on the target, thus relieving the fear of losing face. However, when the targets of negative gossip have a low level of self-monitoring, it is more likely that negative gossip further aggravates their bad reputation, because they lack adequate preparation and suppression skills (Turnley and Bolino, 2001), thus increasing the fear of losing face. As a result,

H5. Self-monitoring moderates the relationship between negative workplace gossip and fear of losing face. The relationship is less positive when targeted employees have a high level of self-monitoring.

H6. Self-monitoring moderates the mediating effect of fear of losing face on the relationship between negative workplace gossip and engagement in negative gossip of the targets, such that the mediating effect is stronger for the targets with low levels of self-monitoring than for those with high levels of self-monitoring.

**Figure 1:**
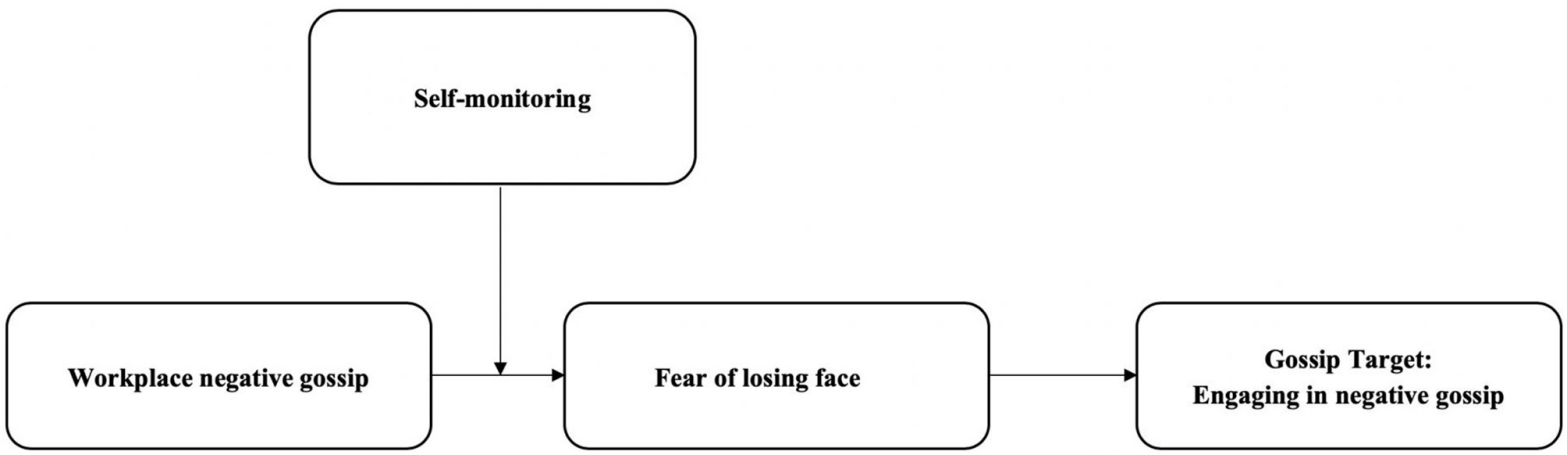
Theoretical model of the current research. Note: Negative workplace gossip and self-monitoring were both measured at time point 1, fear of losing face was measured at time point 2, and engaging in negative gossip was measured at time point 3.

Figure 1 depicts our theoretical framework.

## Methods

### Data and Sample

The participants were recruited from a large entertainment company located in Beijing, China. At time 1 of data collection, we first assured the participants of confidentiality and voluntary participation, and then distributed the questionnaires to a total of 600 participants from the company. Among them, 498 participants (82.67%) returned the questionnaires. We surveyed all the participants at time 1 again a month later (time 2), and 420 valid questionnaires from the 498 participants were returned, representing a response rate of 84.33%. Finally, another month later (time 3), we surveyed the participants who responded at time 2, yielding the final sample of 326 participants (77.6%).

Among the participants, 144 (44.17%) were female, and their average organizational tenure was 4.97 years (sd = 4.36). On average, the participants were 32.95 years of age (sd = 7.79). The majority of the respondents (52.1%) had a bachelor's degree. In terms of potential non-response bias, the response analyses revealed that the individuals in the final sample of 326 employees were not significantly different from those who were dropped from the analyses in terms of demographics and negative workplace gossip measured at T1. We followed the procedures strictly to translate the English-based measures into Chinese (Brislin, 1980). All the surveys conducted for the three time points were in Chinese.

### Measures

We tested the theoretical model across three time points. Specifically, demographic information, engaging in negative gossip, and workplace negative gossip were measured at time 1. A month later (time 2), the participants reported their self-monitoring and fear of losing face. Another month later (time 3), the participants rated their engagement in negative gossip again. Considering the difference between the definition of gossip of a layperson and the theoretical one, we introduced the definition of workplace gossip to the subjects before they filled in the questionnaire in order to avoid misunderstanding of the concept. In particular, we emphasized therein the two basic characteristics of gossip: involving, namely, three parties and the absence of the targets.

#### Negative Workplace Gossip (T1)

This measured being the target of gossip. A five-item scale developed by Brady et al. (2017) was used to measure negative workplace gossip. The items were rated on a seven-point response scale ranging from 1 (never) to 7 (more than once a day), and were preceded by a stem referring to “in the last month, how often have colleagues.” The example items were, “asked a work colleague if they have a negative impression of something that I have done” and “questioned my abilities while talking to another work colleague” (α = 0.96).

#### Fear of Losing Face (T2)

A month later, the participants rated fear of losing face with a five-item scale developed by Zhang et al. (2011). The response options ranged from 1 (strongly disagree) to 7 (strongly agree). The example items include: “for the past month, I have always avoided talking about my weakness,” “for the past month, it was hard for me to acknowledge a mistake, even if I was really wrong,” and “for the past month, I have done my best to hide my weakness before others” (α = 0.87).

#### Self-Monitoring (T2)

We measured self-monitoring of the participants with an 18-item scale developed by Snyder and Gangestad (1986). The items were evaluated on a five-point response scale ranging from 1 (strongly disagree) to 5 (strongly agree). The example items were, “I guess I put on a show to impress or entertain others,” “In different situations and with different people, I often act like very different persons” (α = 0.88).

#### Engaging in Negative Gossip (T3)

This measures the gossip targets themselves engaging in negative gossip. The participants reported their own engagement in negative gossip with a five-item scale developed by Brady et al. (2017). The response options ranged from 1 (never) to 7 (more than once a day). The example items were as follows: “how often have you asked a work colleague if they have a negative impression of something that another co-worker has done,” and “how often have you questioned a co-worker's abilities while talking to another work colleague” (α = 0.93).

#### Control Variables

Consistent with the previous research on gossip, we controlled for gender, age, education, and organizational tenure of the participants, because these factors are related to engaging in gossip. We controlled for gender, because men and women differ in gossip frequency, content, and attitudes (Davis et al., 2018). We also controlled for age, education, and organizational tenure because these factors affect the gossip behaviors of individuals (Kim et al., 2019). In addition, it is worth noting that we measured engaging in negative gossip both at T1 and T3, mainly to control the influence of engaging in negative gossip at T1 on engaging in negative gossip at T3. This is because there may be an upward spiral, as previously noted in the mistreatment literature (e.g., Greco et al., 2019), namely, an abuser is more likely to be retaliated against by others and then engage in more abusive behavior. Therefore, we controlled engaging in negative gossip at T1 in our model.

## Results

### Analytic Strategy

We first conducted a confirmatory factor analysis (CFA) with Mplus 7.0 (Muthen and Muthen, 2012). We then used the SPSS 22.0 software to test the direct effects, and selected Model 7 for PROCESS plug-in to test our moderated mediating model in order to estimate the model and to obtain bias-corrected bootstrapped confidence intervals (using 2,000 bootstrap samples) (Hayes, 2017).

### Descriptive Statistics

Table 1 presents descriptive statistics and correlations of all key variables.

**Table 1:** Descriptive statistics, reliability estimates, and study variable intercorrelations.

**Variables**	**Mean**	**SD**	**1**	**2**	**3**	**4**	**5**	**6**	**7**	**8**	**9**
1. Education (T1)	2.21	0.88									
2. Gender (T1)	3.44	0.49	0.07								
3. Age (T1)	32.95	7.79	−0.08	−0.02							
4. Organizational tenure (T1)	4.97	4.36	−0.01	−0.02	0.54^**^						
5. Engaging in negative gossip (T1)	3.33	1.24	−0.11	−0.02	−0.06	−0.03					
6. Negative workplace gossip (T1)	1.88	1.12	−0.11	−0.02	−0.03	−0.01	0.15	(0.96)			
7. Fear of losing face (T2)	3.93	1.24	−0.14^*^	0.07	0.02	−0.06	0.15^**^	0.45^**^	(0.87)		
8. Self-monitoring (T2)	2.75	0.75	−0.14^*^	−0.05	−0.06	−0.01	0.01	0.4^**^	0.37^**^	(0.88)	
9. Engaging in negative gossip(T3)	1.81	0.74	−0.04	−0.02	−0.04	−0.02	0.32^**^	0.35^**^	0.31^**^	0.18^**^	(0.93)

*N = 326. Cronbach's alphas are shown in parentheses on the diagonal*.

*Gender “1”– male; “2”– female; Education “1”– high school or lower; “2”– junior college; “3”– bachelor's degree; “4”– master's degree or higher*.

*T1, time 1; T2, time 2*.

* *p < 0.05*,

** *p < 0.01*.

### Confirmatory Factor Analyses

Prior to confirmatory factor analyses, we first performed a Harman's single-factor test considering that all the variables in this study, i.e., negative workplace gossip, fear of losing face, self-monitoring, and engaging in negative gossip, were collected from the same source (Podsakoff and Organ, 1986). The result showed that only a factor emerged with only 29.1% of the variance, indicating that the problem of common method bias may be avoided in the current study. Then, we conducted a set of CFAs to further ensure the satisfactory discriminant validity of negative workplace gossip, fear of losing face, self-monitoring, and engaging in negative gossip. The results suggested that the hypothesized four-factor model (χ*2* = 535.44, *df* = 224, CFI = 0.93, TLI = 0.92, RMSEA = 0.04) yielded better fit than any other alternative models (see Table 2).

**Table 2:** Model fit results for confirmatory factor analyses.

**Models**	***χ*^2^**	***df***	**CFI**	**TLI**	**RMSEA**
Hypothesized four-factor model	535.44	224	0.93	0.92	0.04
Three-factor model^a^: NWG and FLF combined	1,194.27	227	0.78	0.75	0.11
Three-factor model^b^: NWG and ENG combined	1,081.1	230	0.81	0.78	0.11
Three-factor model^c^: SM and FLF combined	1,242.88	227	0.77	0.74	0.12
Two-factor model^d^: NWG, FLF, and ENG combined	1,855	232	0.63	0.59	0.15
Single-factor model	2,522.74	230	0.48	0.43	0.18

*N = 326. df, degrees of freedom; CFI, comparative fit index; TLI, Tucker–Lewis index; RMSEA < root-mean-square error of approximation*.

a *Negative workplace gossip and fear of losing face combined*.

b *Negative workplace gossip and engaging in negative gossip combined*.

c *Self-monitoring and fear of losing face combined*.

d *Negative workplace gossip, fear of losing face, and engaging in negative gossip combined*.

### Tests of Hypotheses

We first conducted a hierarchical multiple regression analysis to test the hypotheses. Hypothesis 1 predicted that negative workplace gossip is positively related to engaging in negative gossip of targets. As shown by Model 6 in Table 3, negative workplace gossip was positively and significantly related to engaging in negative gossip (β = 0.35, *p* = 0, *p* < 0.001), supporting Hypothesis 1.

**Table 3:** Results of hierarchical regression analysis.

	**Fear of losing face(T2)**												**Engaging in negative gossip (T3)**														
	***B***	***SE***	**β**	***B***	***SE***	**β**	***B***	***SE***	**β**	***B***	***SE***	**β**	***B***	***SE***	**β**	***B***	***SE***	**β**	***B***	***SE***	**β**	***B***	***SE***	**β**	***B***	***SE***	**β**
	**Model 1**			**Model 2**			**Model 3**			**Model 4**			**Model 5**			**Model 6**			**Model 7**			**Model 8**			**Model 9**		
**Control variables:**																											
Education (T1)	−0.19^*^	0.08	−0.14^*^	−0.12	0.07	−0.09	−0.09	0.07	−0.07	−0.1	0.07	−0.07	−0.03	0.05	−0.04	−0.01	0.04	−0.01	0.01	0.04	0.02	0.01	0.04	0.02	0.01	0.04	0.02
Gender (T1)	0.2	0.14	0.08	0.22	0.12	0.09	0.24^*^	0.12	0.1^*^	0.25^*^	0.12	0.1^*^	−0.03	0.08	−0.02	−0.02	0.08	−0.02	−0.05	0.08	−0.03	−0.05	0.08	−0.03	−0.05	0.08	−0.03
Age (T1)	0.01	0.01	0.07	0.02	0.01	0.09	0.02	0.01	0.11	0.02^*^	0.01	0.12^*^	−0.01	0.01	−0.05	−0.01	0.01	−0.03	−0.01	0.01	−0.05	−0.01	0.01	−0.05	−0.01	0.01	−0.05
Organizational tenure (T1)	−0.03	0.02	−0.1	−0.03	0.02	−0.11	−0.03^*^	0.02	−0.12^*^	−0.03^*^	0.02	−0.11^*^	0.01	0.01	0.01	0	0.01	0	0	0.01	0.02	0	0.01	0.02	0	0.01	0.02
Engaging in negative gossip (T1)	0.16	0.05	0.16	0.09	0.05	0.09	0.15	0.05	0.15	0.14	0.05	0.14	0.2^**^	0.03	0.33^**^	0.17^**^	0.03	0.29^**^	0.16^**^	0.03	0.27^**^	0.16^**^	0.03	0.28^**^	0.17^**^	0.03	0.28^**^
**Independent variable:**																											
Negative workplace gossip (T1)				0.5^***^	0.06	0.45^***^	0.34^***^	0.06	0.36^***^	0.48^***^	0.07	0.43^***^				0.23^***^	0.04	0.35^***^	0.18^***^	0.04	0.27^***^	0.18^***^	0.04	0.26^***^	0.17^***^	0.05	0.26^***^
**Mediator:**																											
Fear of losing face (T2)																			0.12^**^	0.04	0.19^**^	0.11^**^	0.04	0.19^**^	0.12^**^	0.04	0.19^**^
**Moderator:**																											
Self-monitoring (T2)							0.38	0.09	0.23	0.4	0.09	0.24										0	0.06	0	0	0.06	0
**Interaction:**																											
Negative workplace gossip ×										−0.13^*^	0.06	−0.13^*^													0	0.04	0
× self-monitoring																											
*R*^2^		0.18			0.48			0.27			0.28			0.01			0.13			0.15			0.15			0.15	
Δ*R*^2^		0.02			0.2			0.04			0.01			0.01			0.12			0.03			0.01			0.01	
*F*	2.71^*^	18.89^***^	19.8^***^	17.8^***^	0.3	9.12^***^	9.62^***^	8.22^***^			7.17^***^																

*N = 326*.

* *p < 0.05*;

** *p < 0.01*;

*** *p < 0.001*.

*T1, time 1; T2, time 2*.

*R^2^, Effect size, which reflects the proportion of all variations in the dependent variable that can be explained by the independent variable*.

Hypothesis 2 predicted that negative workplace gossip is positively related to fear of losing face. As shown by Model 2 in Table 3, negative workplace gossip was positively related to fear of losing face (β = 0.45, *p* = *0.0*01, *p* < *0.01*), supporting Hypothesis 2.

As indicated by Model 7 in Table 3, fear of losing face was positively related to engaging in negative gossip (β = 0.19, *p* = *0.0*01*, p* < 0.01). Therefore, Hypothesis 3 was supported.

Then, for generating bias-corrected 95% confidence intervals (CIs), we employed the PROCESS analysis and opted for Model 7 to test our mediating and moderated mediating effects (Hayes, 2017). Hypothesis 4 predicted that fear of losing face mediates the relationship between negative workplace gossip and engaging in negative gossip. As shown in Table 4, negative workplace gossip was positively related to fear of losing face (β = 0.48, *p* = *0.0*001, *p* < 0.001, bias-corrected bootstrap 95% CI = [0.34, 0.61], and fear of losing face was positively related to engaging in negative gossip (β = 0.12, *p* = *0.0*01*, p* < 0.01, bias-corrected bootstrap 95% CI = [0.05, 0.18]). Additionally, the PROCESS analysis results showed that there was a significant mediation effect through fear of losing face in the relationship between negative workplace gossip and engaging in negative gossip (β = 0.05, bias-corrected bootstrap 95% CI = [0.02, 0.09]). Thus, Hypothesis 4 was supported (also see Table 5 for the regression results without control variables and Table 6 for supplementary results of the moderated path analysis).

**Table 4:** Regression results for moderation and moderated mediation model (bootstrapping).

	**Fear of losing face (T2)**			**Engaging in negative gossip (T3)**		
	**Effect**	**LLCI**	**ULCI**	**Effect**	**LLCI**	**ULCI**
Constant	3.35^***^	2.64	4.07	1.53^***^	1.01	2.05
Education (T1)	−0.09	−0.23	0.04	0.01	−0.07	0.1
Gender (T1)	0.25^*^	0.02	0.49	−0.05	−0.2	0.1
Age (T1)	0.02^*^	0.01	0.04	−0.01	−0.02	0.01
Organizational tenure (T1)	−0.03^*^	−0.06	−0.01	0.01	−0.02	0.02
Engaging in negative gossip (T1)	0.11	−0.01	0.2	0.16^**^	0.11	0.23
Negative workplace gossip (T1)	0.48^***^	0.34	0.61	0.18^***^	0.1	0.25
Fear of losing face (T2)				0.12^**^	0.05	0.18
Self-monitoring (T2)	0.39	0.22	0.56			
Negative workplace gossip × self-monitoring	−0.12^*^	−0.24	−0.01			
	**Conditional statistical results**			**Conditional indirect statistical results**		
Self-monitoring M – SD	0.57^***^			0.06	0.02	0.10
Self-monitoring M + SD	0.38^***^			0.04	0.02	0.08
Differences between low and high	−0.21^***^			−0.02	−0.05	−0.01

*N = 326. Boot LCI, bootstrapped lower confidence interval; Boot UCI, bootstrapped upper confidence interval; β, represents standardized path coefficients; bootstrap sample size = 2,000*.

*T1, time 1; T2, time 2*.

* *p < 0.05*;

** *p < 0.01*;

*** *p < 0.001*.

**Table 5:** Supplementary regression results (without control variables).

	**Fear of losing face**			**Engaging in negative gossip (T3)**		
	**Effect**	**LLCI**	**ULCI**	**Effect**	**LLCI**	**ULCI**
Negative workplace gossip	0.47^***^	0.34	0.61	0.18^***^	0.1	0.25
Fear of losing face				0.11^**^	0.04	0.18
Self-monitoring	0.39	0.22	0.56			
Negative workplace gossip × self-monitoring	−0.12^*^	−0.24	0.001			
**Conditional indirect statistical results**				**Effect (*****R***^**2**^**)**	**LLCI**	**ULCI**
Self-monitoring *M – SD*				0.06 (0.22)	0.02	0.10
Self-monitoring *M + SD*				0.04 (0.30)	0.02	0.70
Differences between low and high				−0.02 (0.34)	−0.04	−0.002

*N = 326. Boot LCI, bootstrapped lower confidence interval; Boot UCI, bootstrapped upper confidence interval; β, represents standardized path coefficients; bootstrap sample size = 2,000*.

*T1, time 1; T2, time 2*.

*R^2^, Effect size, which reflects the proportion of all variations in the dependent variable that can be explained by the independent variable*.

* *p < 0.05*;

** *p < 0.01*;

*** *p < 0.001*.

**Table 6:** Supplementary results of the moderated path analysis.

**Moderator variable**	**Negative workplace gossip (X)**→**Fear of losing face (M)**→**Engaging in negative gossip (Y)**				
	**Stage**		**Effect**		
	**First**	**Second**	**Direct effects**	**Indirect effects**	**Total effects**
	**(P_**MX**_)**	**(P_**YM**_)**	**(P_**YX**_)**	**(P_**YM**_P_**MX**_)**	**(P_YX_+ P_YM_P_MX_)**
Simple paths for low self-monitoring	−0.12^*^	0.07	0.07	0.06^*^	0.13
Simple paths for high self-monitoring	−0.13^*^	0.05	−0.01	0.04^*^	0.03^*^
Differences	−0.01^*^	−0.02	−0.08	−0.02^*^	−0.1

*P_MX_, Path from negative workplace gossip to fear of losing face; P_YM_, Path from fear of losing face to engaging in negative gossip; P_YX_, Path from negative workplace gossip to engaging in negative gossip. Low self-monitoring refers to one standard deviation below the mean value of self-monitoring. High self-monitoring refers to one standard deviation above the mean value of self-monitoring*.

*Tests of differences for direct, indirect, and total effects were based on bias-corrected confidence intervals obtained from bootstrapping estimates*.

*The above coefficients are standardized coefficients*.

* *p < 0.05*.

**Figure 2:**
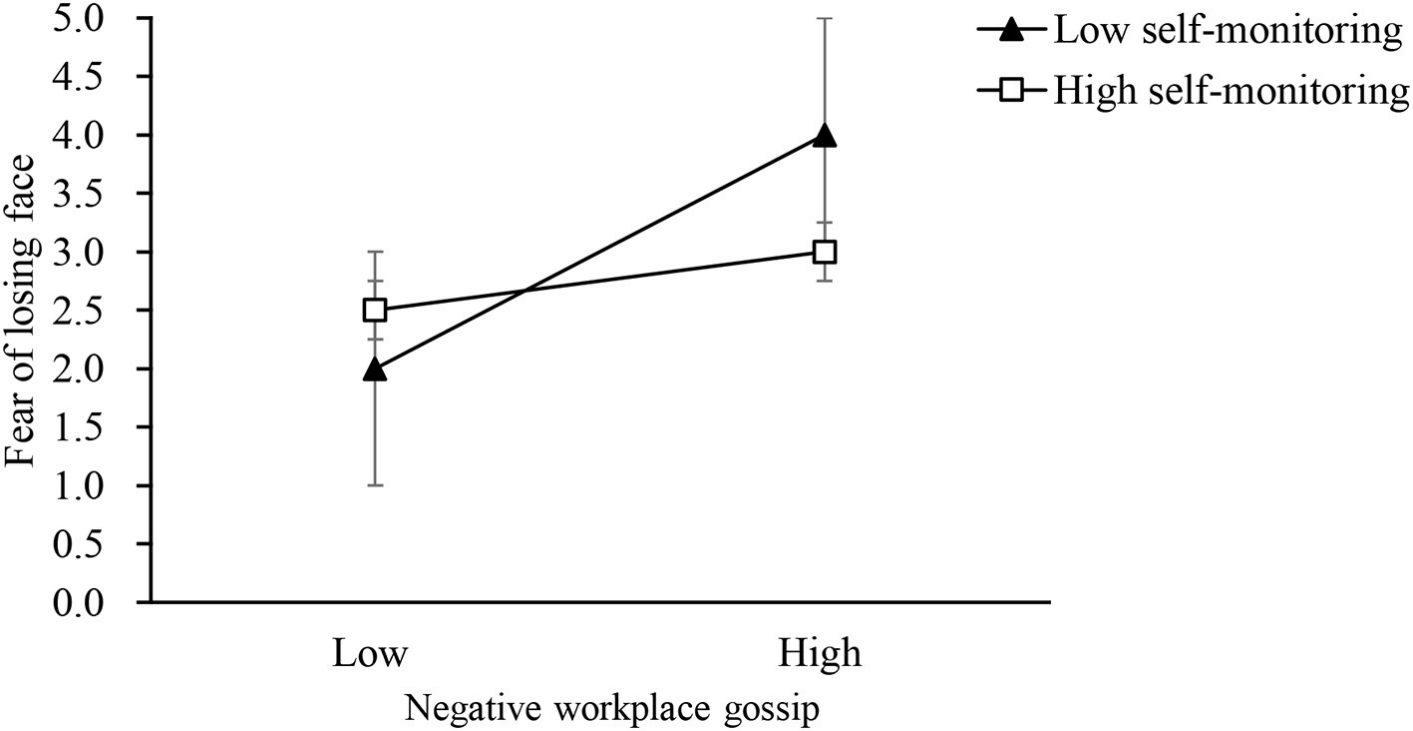
The moderating role of self-monitoring on the relationship between negative workplace gossip and fear of losing face. Note: High and low levels represent 1 SD above and below the mean, respectively. The error bars are represented around the points.

Regarding the moderating effects in this study, Hypothesis 5 predicted that self-monitoring moderates the relationship between negative workplace gossip and fear of losing face. As shown by Model 4 in Table 3, the interaction between negative workplace gossip and self-monitoring was negatively related to fear of losing face (β = −0.12, *SE* = 0.06, *p* = 0.036, *p* < 0.05). Using the procedure of Aiken et al. (1991), we plotted the relationship between negative workplace gossip and fear of losing face according to two levels of self-monitoring, namely, one standard deviation above the mean and one standard deviation below the mean. Figure 2 shows that the positive effect between negative workplace gossip and fear of losing face was stronger when self-monitoring was low (simple slope = 0.57, *SE* = 0.1, *p* < 0.001) rather than high (simple slope = 0.38, *SE* = 0.06, *p* < 0.001), supporting Hypothesis 5.

Finally, the results presented in Table 4 provided empirical support for Hypothesis 6. The results showed that self-monitoring moderated the mediating effect of fear of losing face on the relationship between negative workplace gossip and engaging in negative gossip of the targets, such that the mediating effect was stronger for targets with low levels of self-monitoring than for those with high levels of self-monitoring. Specifically, the mediated relationship between negative workplace gossip and engaging in negative gossip through fear of losing face was stronger when self-monitoring was low (i.e., conditional mediation effect = 0.07, 95% CI = [0.03, 0.11]) vs. high (i.e., conditional mediation effect = 0.04, 95% CI = [0.02, 0.08]). The difference between the two conditions was −0.02 with 95% CI [−0.05, −0.001]. Thus, we obtained support for Hypothesis 6.

## Discussion

### Theoretical and Practical Implications

Based on face research and AET, this study developed theoretical arguments and empirically tested the relationships between being the target of negative gossip and engaging in negative gossip behavior of the targets, and further explored the role of face as a mediating mechanism in this process, namely, fear of losing face. This study also examined a contingent effect, namely, self-monitoring, which mitigated the positive relationship between negative workplace gossip and fear of losing face. The results supported the hypotheses. In the following, we discuss how our findings contribute to the existing literature and managerial practices.

This study contributes to prior studies by investigating how targets respond to negative workplace gossip from the perspective of face. As such, we introduced a novel mediating mechanism to the gossip literature, fear of losing face. The findings showed that negative workplace gossip, as an affective event, could enhance fear of losing face and thereby trigger engaging in negative gossip of the targets. This study makes three contributions to the study on negative gossip. First, we add to the research on target responses to gossip. There have been some negative gossip studies that investigated the behavioral responses of the targets after hearing gossip about them. In particular, these studies have focused either on passive responses of the targets, such as a reduction in extra performance or discretionary behavior (Wu X. et al., 2018) and even turnover intentions (Brady et al., 2017), or on adaptive responses, such as cooperation behavior that arises from the concern for reputation or the need to integrate into a group (e.g., Feinberg et al., 2014; Dores Cruz et al., 2019b, 2020). However, relatively less attention has been paid to the active responses of the targets, such as gossiping. We do acknowledge that gossip research has already theorized that the targets of gossip may have a tendency to cope actively. However, in fact, few empirical studies have specified which specific detrimental behavior that the targets may engage in against the perpetrators, let alone studies that have directly examined the relationship between the two. In this study, we empirically found that the gossip targets themselves would also participate in gossip, acting as gossip senders, and theoretically explicated the process by which the gossip targets became gossip senders themselves. In this regard, we extend past studies on the responses of gossip targets. In addition, in terms of the positive aspects of gossip, scholars have pointed out that sharing of negative gossip about a target can have a pro-social motive and be used as a means for effectively deterring selfishness and promoting cooperation of the target, such as protecting others from antisocial or exploitative behavior (e.g., Feinberg et al., 2012, 2014; Milinski, 2016; Dores Cruz et al., 2019a, 2020). However, the results indicated that the targets of negative gossip were indeed likely to deal with the gossipers actively rather than merely respond in a cooperative or adaptive manner. On this basis, we encourage future research to explore the conditions under which gossip targets are more likely to respond in a passive, adaptive, or active way, or in combination of all three.

Second, we provide a faced-based mediating mechanism for workplace gossip research. Specifically, we identified fear of losing face as a mediating mechanism between negative workplace gossip and engaging in negative gossip. Past research has provided some important theoretical perspectives on the effects of gossip on targets, such as social exchange theory (Lee et al., 2016), emotion-related theory (Wu X. et al., 2018), social identity theory (Ye et al., 2019), and conservation of resource theory (Cheng et al., 2020), all of which significantly advance the research on gossip. These perspectives effectively reveal the negative effects of workplace gossip on emotions, cognitions, and behaviors of the targets. For example, negative gossip leads to emotional exhaustion and negative mood (Wu X. et al., 2018; Babalola et al., 2019), increases ego depletion and organization-based self-esteem (Wu L. Z. et al., 2018; Cheng et al., 2020), and reduces organizational citizenship behavior, innovative behavior, and other extra-role behaviors (Wu L. Z. et al., 2018; Wu X. et al., 2018; Zhou et al., 2019). In contrast, based on a face-based perspective, this study focused more on how these gossip targets dealt with the perpetrators in an active manner, e.g., gossiping behavior, to regain their face. Negative gossip can be insidious and undetectable, and differs from mistreatments such as abuse, workplace bullying, workplace ostracism, and physical aggression (Duffy et al., 2002). Given that face is related to the assessment of abilities of someone in a position, people may be more likely to take relatively covert and safe actions than take a risk with offensive actions, which would do more damage to the face of someone. Further, there is a subtle difference between face and reputation, although the latter also suggests that the targets of gossip may engage in reputation-seeking behavior because of reputation concerns, such as cooperative and organizational citizenship behavior (e.g., Piazza and Bering, 2008; Beersma and Van Kleef, 2011; Wu L. Z. et al., 2018). By definition, face represents the self-worth of a person gained by performing specific social roles that are well-recognized by others (Ho, 1976), whereas reputation reflects the observable qualities or attributes of a person (e.g., gender, age education, institution granting degrees, experience) (Spence, 1974; Ferris et al., 1994). This means that people who perform well in their position, even if they have a bad reputation, will gain face. In this regard, face may be more directly associated with workplace gossip, since workplace gossip mainly involves the evaluation of work-related aspects, while reputation is more multisource and characterized primarily by personal qualities. Thus, compared with reputation theoretically elaborated in previous studies, this study considers fear of losing face as the mediating mechanism of gossip to provide more detailed understanding of how gossip in organizations affects the response of the target.

Finally, the moderating effect of self-monitoring showed that it is a boundary mechanism for how targets react to workplace gossip. Specifically, our findings showed that high self-monitors not only relieved the detrimental effect of negative workplace gossip on them, they also reduced their own gossiping. Therefore, this result, on one hand, verifies the view of previous scholars that self-monitoring, as a personality trait, could be used to effectively deal with workplace gossip (e.g., Xie et al., 2019); on the other, it also provides us with insights, that is, it may be able to restrain the targets from engaging in negative gossip. Existing studies on the boundary mechanisms of gossip have generally focused on the following aspects: situational characteristics, e.g., organizational change, uncertainty, and ambiguity (Mills, 2010); job social support (Tian et al., 2019); work-unit cohesiveness (Loughry and Tosi, 2008); civility climates (Li et al., 2019), gossip characteristics, e.g., gossip veracity (Dores Cruz et al., 2019a); statue of target (Ellwardt et al., 2012); relationships in gossip triad; content of gossip (Tassiello et al., 2018; Giardini and Wittek, 2019), cognitions, e.g., traditionality (Wu X. et al., 2018); just world beliefs (Zhou et al., 2020); reputational concerns (Martinescu et al., 2019a,b); creative self-efficacy (Zhou et al., 2019); trustworthiness (Lee and Barnes, 2020); perceived insider status (Kim et al., 2019), and emotions, e.g., negative affectivity (Wu L. Z. et al., 2018), all of which have made outstanding contributions to the boundary mechanisms of gossip. However, at the same time, we notice that the research on personality traits is still insufficient. In fact, personality traits may have a great potential to play a role in the influence process of gossip. For example, the dark triad (i.e., psychopathy, Machiavellianism, and narcissism) is characterized by callousness and a tendency to manipulate others for own benefit (Jones and Figueredo, 2013). It may be more resistant to gossip about themselves, and thus may provide valuable insights for gossip research. To address this gap, we encourage future research to introduce more relevant personality traits and explore their roles in gossiping.

This study has two important practical implications. First, it shows that the targets of negative gossip engaged in negative gossip for fear of losing face. The findings remind managers to pay special attention to employee face issues. Personal face is related to the competence and reputation of someone in a position, which is related to the personal status of an employee in a group and future career development; thus, employees often attach great importance to it. It is a prevalent yet easily overlooked phenomenon in organizational management. Indeed, we rarely see provisions on face in official documents or daily regulations of organizations. However, the findings of this study show that employees who were concerned about losing face would respond to the perpetrators to seek revenge, eventually having a negative impact on individuals and organizations. Therefore, we believe that managers should pay more attention to the face needs of employees. For example, employees who have performed well in their position should be praised publicly and ceremonially. In addition, we also suggest that organizations should establish effective formal feedback channels or communication mechanisms, rather than rely solely on the own gossip networks of employees, in order to help employees communicate with each other about work-related information and provide emotional counseling. Second, our conclusions indicate that self-monitoring might be a personality trait that effectively responds to negative gossip, and that it might also be able to inhibit the subsequent gossip behavior of someone. This conclusion especially reminds us to pay more attention to people with low self-monitoring in the workplace, because they may be more likely to suffer from the negative effects of negative workplace gossip. In this regard, we suggest that managers could provide employees with a variety of training programs on interpersonal communication, conflict, and psychological construction while allowing them to make their own choices, so as to improve their ability to deal with possible gossip about them and other complex interpersonal relationships.

### Limitations

There are still several limitations. First, reliance on self-report data in the research may raise concerns about common method variance, but this practice has been inherent in past studies on perceptions of gossip, or in other similar measures that reflect changes in the internal state and behavior of actors. This is because the primary focus of this study was on perceptions of gossip targets rather than on actually having been the targets of gossip, and subsequent emotional and behavioral consequences. In this way, we cannot fully assess whether the perceptions of gossip about them are subjective or, in fact, have already occurred in this group. However, past research has shown that targets of gossip often know who gossiped about them. Predominantly they had heard about the gossip from the recipients, or they had learned about it accidentally (Martinescu, 2017; Dores Cruz et al., 2019a). Nevertheless, addressing this, future studies are encouraged to collect data from multiple sources and reexamine the hypotheses of this study. Similarly, the outcome variable, engaging in negative gossip, was self-reported. Thus, we also encourage reporting from multiple sources in the future.

Second, we collected three-wave data, which do not allow causal inference. Despite that, the research model is validated theoretically and empirically. Longitudinal studies and field experiments are still necessary, considering that they are more effective in causality. In this study, we predicted that being the target of gossip would lead to more gossiping itself. Nevertheless, we cannot rule out that it was the gossiping of the targets that made them the target of gossip later on. Based on this, we encourage future studies to reexamine these relationships in longitudinal or experimental ways to further clarify the causation between them.

Finally, in our questionnaires, the word gossip may have negative connotations. Indeed, both a lay perspective and the available data seem to bear this out (e.g., a Dutch sample, Dores Cruz et al., 2020). Therefore, we encourage future researchers to take this into account and rule out the disturbing effects of this problem on their studies. For example, researchers could write a brief description at the top of the questionnaire to inform the participants of the theoretical definition of gossip.

## Conclusion

Workplace gossip is a common phenomenon within organizations. Based on face research and AET, this research explored the mediating role of fear of losing face between negative workplace gossip and engagement in negative gossip of the targets and further included self-monitoring as a regulating factor. The results supported the hypotheses. Considering that the information contained in gossip is closely related to the parties concerned, we call for more research on gossip in the workplace and its impact on targets.

## Data Availability Statement

The raw data supporting the conclusions of this article will be made available by the authors, without undue reservation.

## Ethics Statement

The studies involving human participants were reviewed and approved by Ethics Committee of Renmin University of China. The patients/participants provided their written informed consent to participate in this study.

## Author Contributions

BZ, SX, and LZ: conceptualization. BZ and JQ: methodology. BZ: writing original draft. BZ, SX, LZ, and JQ: review and editing. All authors contributed to the article and approved the submitted version.

## Conflict of Interest

The authors declare that the research was conducted in the absence of any commercial or financial relationships that could be construed as a potential conflict of interest.
